# Discovery of a novel sub-lineage of multi-drug resistant *Shigella flexneri* in Southern California

**DOI:** 10.1016/j.ijid.2023.03.039

**Published:** 2023-03-28

**Authors:** Edwin Kamau, Paul C. Adamson, John Crandall, Rituparna Mukhopadhyay, Shangxin Yang

**Affiliations:** 1 Department of Pathology and Laboratory Medicine, University of California Los Angeles, David Geffen School of Medicine, Los Angeles, California; 2 Division of Infectious Diseases, University of California Los Angeles, David Geffen School of Medicine, Los Angeles, California; 3 Microbial Diseases Laboratory, California Department of Public Health, Richmond, California

**Keywords:** Multi-drug resistant Shigella, Shigella flexneri, Men who have sex with men, Whole genome sequencing

## Abstract

Clustered outbreaks of multi-drug resistant (MDR) *Shigella* are on the rise among men who have sex with men (MSM). Identification of MDR sub-lineages is critical for clinical management and public health interventions. Here, we describe a novel MDR sub-lineage of *Shigella flexneri* isolated from an MSM patient without a travel history in Southern California. Detailed genomic characterization of this novel strain would serve as a reference to aid monitoring and future outbreak investigation of MDR *Shigella* among MSM.

## Short communication

Outbreaks of sexually transmitted *Shigella* among men who have sex with men (MSM) are frequently reported [1,2] and multi-drug resistant (MDR) *Shigella* among MSM are of particular concern [2–4]. The first-line treatment for domestically acquired shigellosis in the US is fluoroquinolone or azithromycin, depending on local susceptibilities or travel history [5]. Other treatment options include ceftriaxone and trimethoprim-sulfamethoxazole (TMP-SMX) based on antimicrobial susceptibility testing (AST) phenotypic data. In addition to mutations in genes encoding antibiotic targets, other genomic drivers conferring antimicrobial resistance (AMR) in *Shigella* are in mobile elements within the genomes and/or plasmids facilitating transmission, acquisition, and development of AMR [6,7]. Here, we describe a novel MDR sub-lineage of *Shigella flexneri* linked to sexual transmission among MSM in Southern California.

A 60-year-old man with well-controlled HIV-1 (undetectable viral load, cluster of differentiation 4 count of 959) presented to the emergency department (ED) of another hospital with watery diarrhea and abdominal pain. He reported having several recent male sex partners in the preceding 2 weeks, at least one of whom subsequently developed diarrhea. He was empirically treated with a 3-day course of azithromycin. Three weeks later, he was seen in our hospital ED after developing a new episode of diarrhea with blood and mucus, following 3 days of abdominal pain and chills. He denied any sexual activity in that period. He was empirically started on ciprofloxacin (500 mg PO BID) and a stool specimen was submitted to the laboratory for bacterial enteric pathogen panel polymerase chain reaction (PCR) testing (BD MAX^™^ Enteric Bacterial Panel). On day 4, ciprofloxacin was increased to 750 mg PO BID given ongoing symptoms and based on treatment recommendations among people living with HIV [8]. PCR testing returned positive for *Shigella*, automatically triggering a culture work-up to isolate the bacteria for definitive species identification and AST. By day 7, the patient’s symptoms had improved. However, AST results revealed the patient was infected with an MDR *S. flexneri,* resistant to ciprofloxacin and azithromycin (Table 1). Treatment was switched to 3 days of cefpodoxime (400 mg PO BID), due to insurance issues and because the patient was not available for injectable antibiotics; this was followed by ceftriaxone 2 g intravenously every 24 hours for 5 days.

The isolate (UCLA689) was sequenced on MiSeq (Illumina, California, US) using 2 × 250 bp protocol. Whole-genome sequencing (WGS) analysis was performed using CLCbio genomics workbench (QIAGEN, California, US) and Geneious Prime software (Biomatters, New Zealand). Phylogenetic studies were performed using a previously validated method [3]. A custom shell script automated the subsequent steps by taking the mapped files through a series of software suites that included: SAMtools mpileup (v.1.2) calculating the genotype likelihood; bcftools (v0.1.19) converting into variant call format (VCF) matrix and calling single nucleotide polymorphism (SNP) in coding and non-coding regions; vcftools (v.0.1.12b) to parse and only include high-quality SNPs (hqSNPs) excluding any InDels or heterozygote call; Perl Script to convert SNP matrix into FASTA alignment file for CLCbio to generate a maximum likelihood phylogenetic tree. AMR genes and plasmid replicons were identified using ResFinder and PlasmidFinder (https://cge.cbs.dtu.dk/services). UCLA689 WGS data were deposited to National Center for Biotechnology Information (NCBI) Sequence Read Archive (SRA) (BioProject ID PRJNA797477).

**Figure 1.**
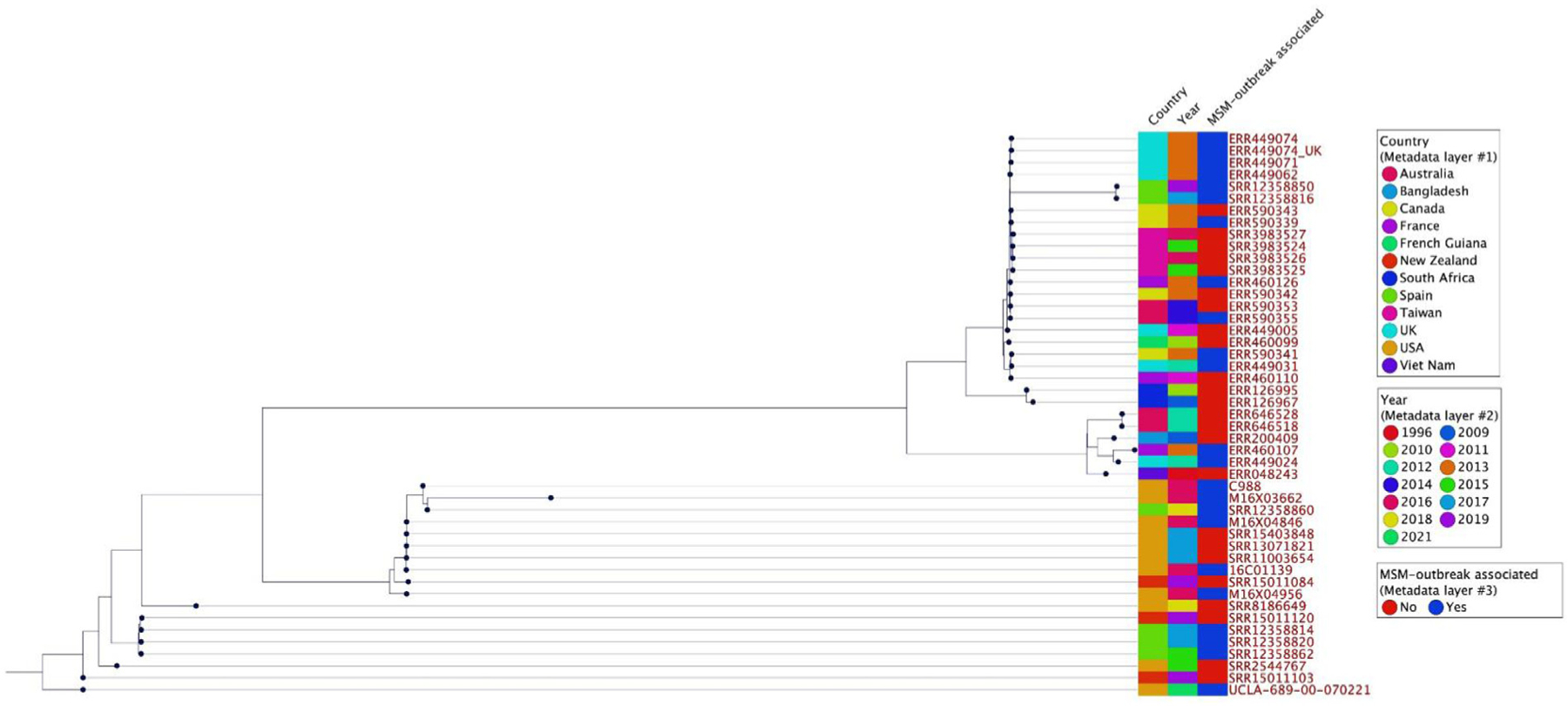
Phylogenetic tree showing the relationship of UCLA689 to *Shigella flexneri* isolates from different countries between 1996 and 2019 and their association with MSM outbreak. MSM, men who have sex with men.

When aligned against the reference *S. flexneri* type 2a American Type Culture Collection (ATCC) strain 700930 (GenBank: AE014073), UCLA689 WGS coverage and pairwise identity (PI) were 99.9% and 98.9%, respectively. The isolate carried a common chromosomal mutation in the *gyrA* (D87N and S83L) gene conferring resistance to quinolones, and additional rare or novel mutations in the *gyrA* (H211Y), *parC* (S80I) and *parE* (S458A) genes (Table 1). Since the clinical isolate before the fluoroquinolone treatment was not available for genomic comparison, it is unclear whether these mutations were present in the original bacterial strain or accumulated during the treatment. The isolate also contained a plasmid highly identical to reference plasmids (pMHMC-004 [GenBank: CP053755] and pKSR100 strain SF7955 [GenBank: LN624486]) with sequence coverage >90% and PI ∼99%, which carried macrolide (*ermB* and *mphA*), TMP-SMX (*dfrA1, dfrA14, sul2*), and beta-lactam (*bla_TEM-1B_*) resistance genes. In addition, tetracycline (*tet(B)*), chloramphenicol (*catA1*), and beta-lactam (*blaOXA-1*) resistance genes were also detected, but their exact locations (i.e., chromosome vs plasmids) were unclear. To infer phylogenetic relationships between UCLA689 and isolates collected during the 2016–2017 *S. flexneri* outbreaks in Southern California, we created an SNP tree. Isolate UCLA689 was phylogenetically different from the outbreak isolates (Figure S1), with ∼2000 SNP difference (Figure S2). Most outbreak isolates (29/33) had <25 SNP difference, indicating they belong to the same sub-lineage. We further compared our isolates with representative genomes previously studied from different countries, years, and associations with known MSM outbreaks [2,3,9]. The phylogenetic tree revealed that isolate UCLA681 was not related to any of the published sub-lineages, with the genetically closest isolate collected in New Zealand in 2019, which was not associated with an MSM outbreak (Figure 1 & Figure S3).

In conclusion, we report a novel MDR *S. flexneri* sub-lineage that was domestically acquired through sexual transmission in an MSM patient. Transmission of MDR shigellosis is a growing concern in the MSM community and may pose an increased risk for those living with HIV [10]. Driven by the presence of plasmids and mobile genetic elements, *Shigella* spp. can acquire and develop antibiotic resistance quickly, which may also spread to non-MSM patient populations. Healthcare providers should consider the possibility of antibiotic resistance in shigellosis cases with poor drug treatment responses. Increased surveillance of shigellosis including genetic and antibiotic susceptibility testing is warranted to better inform clinical management, antimicrobial stewardship, and public health interventions.

## Supplementary Material

Supplement

## Figures and Tables

**Table 1:** Phenotypic drug susceptibility results and genetic resistance markers.

Drug class	Drug	MIC	Interpretation^a^	Resistance marker
Aminoglycoside	Gentamicin	*>*16	Resistant	*acc(3)-IId*
Beta-lactam	Ampicillin	*>*32	Resistant	*blaTEM-1B, blaOXA-1*
	Ceftriaxone	≤1	Susceptible	Not detected
	Ceftazidime	≤0.5	Susceptible	Not detected
	Meropenem	≤0.25	Susceptible	Not detected
	Ertapenem	≤0.25	Susceptible	Not detected
	Piperacillin-Tazobactam	≤8	Susceptible	*blaOXA-1*
Fluoroquinolone	Ciprofloxacin	*>*4	Resistant	*gyrA* mutations (S83L, D87N, **H211Y**) *parC* mutation (**S80I**) *parE* mutations (**S458A**)
	Levofloxacin	*>*8	Resistant	
Folate antagonist	Trimethoprim-sulfamethoxazole	*>*4/80	Resistant	*dfrA1, dfrA14, sul2*
Macrolide	Azithromycin	*>*32	Resistant	*mphA, erm(B)*
Tetracycline	Testing not performed			*tet(B)*
Chloramphenicol	Testing not performed			*catA1*

a MIC interpretations were based on the CLSI breakpoints.Shown in bold are the rare or new mutations described in this study.CLSI, Clinical and Laboratory Standards Institute; MIC, minimum inhibitory concentration.
