# Supplementary material for: Discovery of a novel sub-lineage of multi-drug resistant Shigella flexneri in Southern California
Source: Int J Infect Dis. Author manuscript; Available in PMC 2023 Jul 1. (PMC10275642; doi:10.1016/j.ijid.2023.03.039)

**Supplementary data**

**Figure S1**: Phylogenetic tree of *S. flexneri* isolates collected from Southern California outbreak in 2016-17 and recent *S. flexneri* isolate UCLA-689.


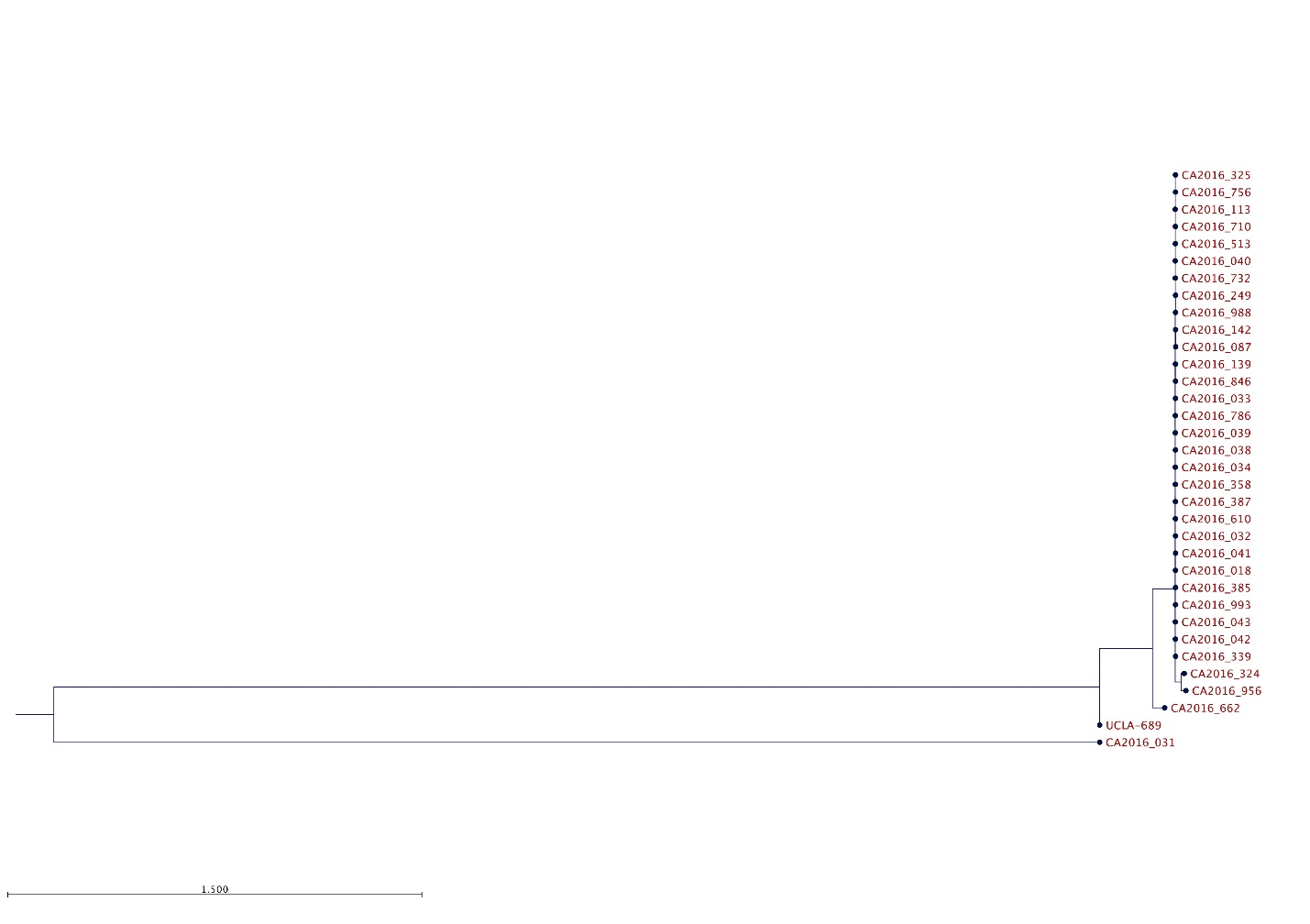


**Figure S2:** Distance matrix of *S. flexneri* isolates collected from Southern California outbreak in 2016-17 and recent *S. flexneri* isolate UCLA-689 showing SNP difference between each isolate.


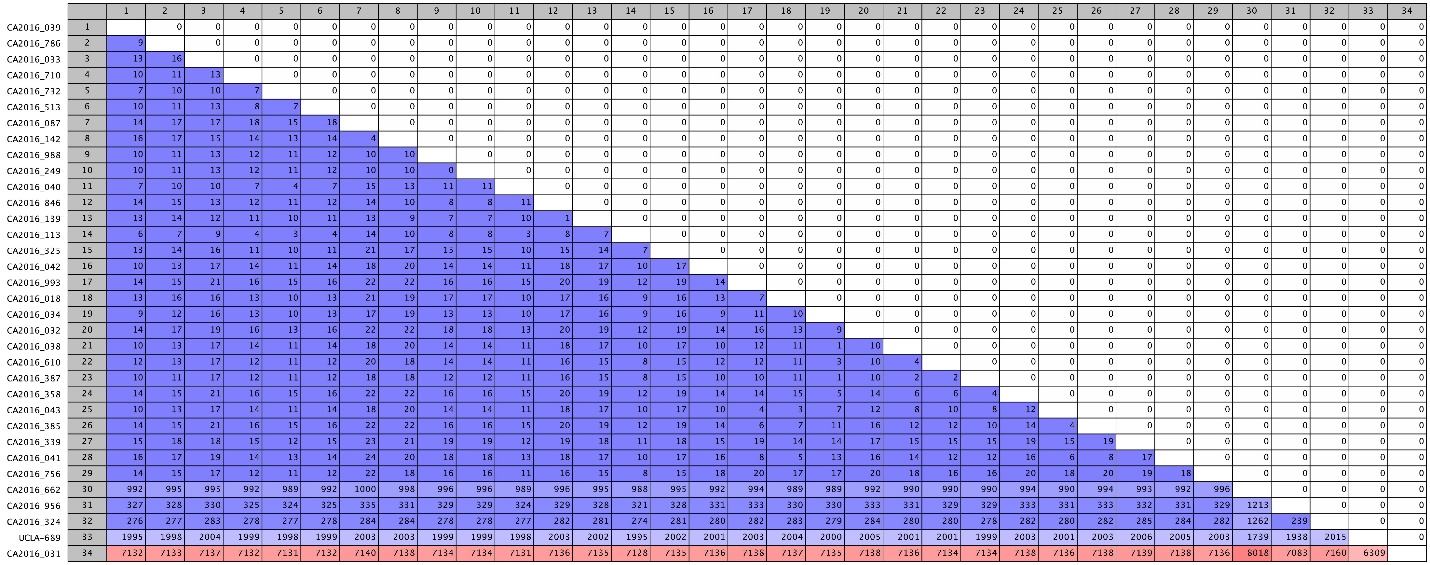


**Figure S3**: Distance matrix showing SNP difference between *S. flexneri* isolate UCLA-689 and *S. flexneri* isolates from different countries between 1996 and 2019 and their association with MSM outbreak.


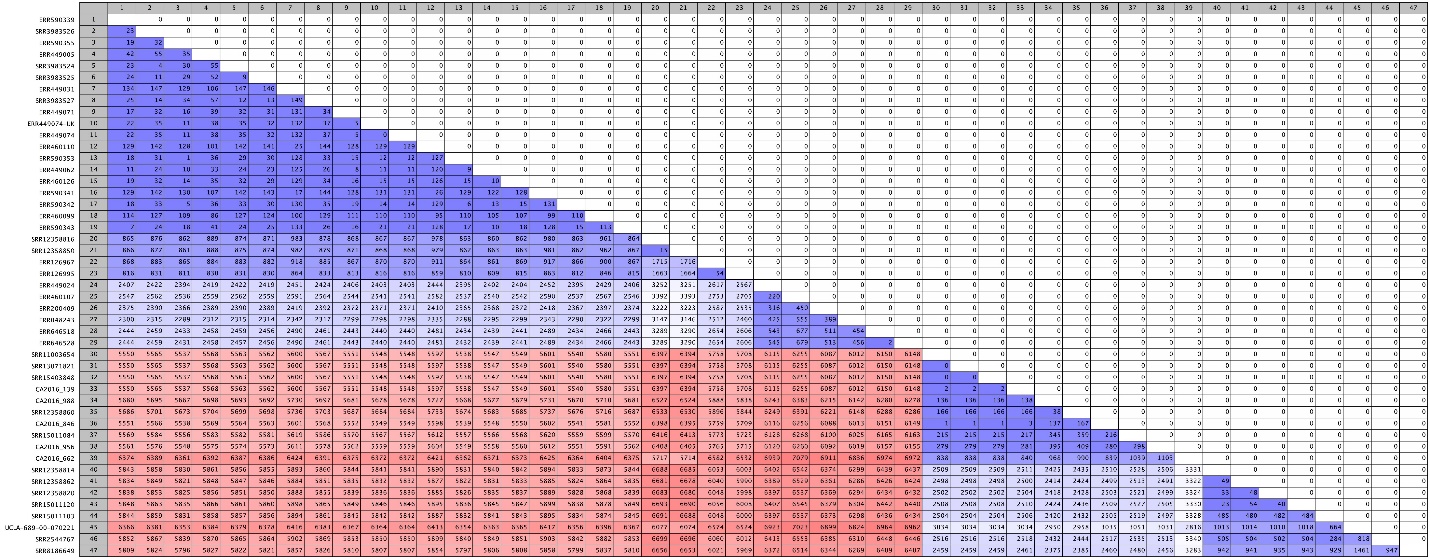

Supplement: Supplement [file NIHMS1904418-supplement-Supplement.docx]
